# Motor Learning: An Analysis of 100 Trials of a Ski Slalom Game in Children with and without Developmental Coordination Disorder

**DOI:** 10.1371/journal.pone.0140470

**Published:** 2015-10-14

**Authors:** Bouwien C. M. Smits-Engelsman, Lemke Dorothee Jelsma, Gillian D. Ferguson, Reint H. Geuze

**Affiliations:** 1 Faculty of Kinesiology and Rehabilitation Sciences, KU Leuven, Gebouw De Nayer (GDN), Heverlee, Belgium; 2 Department of Health and Rehabilitation Sciences, University of Cape Town, Cape Town, South Africa; 3 Developmental and Clinical Neuropsychology, University of Groningen, Groningen, the Netherlands; Durham University, UNITED KINGDOM

## Abstract

**Objective:**

Although Developmental Coordination Disorder (DCD) is often characterized as a skill acquisition deficit disorder, few studies have addressed the process of motor learning. This study examined learning of a novel motor task; the Wii Fit ski slalom game. The main objectives were to determine: 1) whether learning occurs over 100 trial runs of the game, 2) if the learning curve is different between children with and without DCD, 3) if learning is different in an easier or harder version of the task, 4) if learning transfers to other balance tasks.

**Method:**

17 children with DCD (6–10 years) and a matched control group of 17 typically developing (TD) children engaged in 20 minutes of gaming, twice a week for five weeks. Each training session comprised of alternating trial runs, with five runs at an easy level and five runs at a difficult level. Wii scores, which combine speed and accuracy per run, were recorded. Standardized balance tasks were used to measure transfer.

**Results:**

Significant differences in initial performance were found between groups on the Wii score and balance tasks. Both groups improved their Wii score over the five weeks. Improvement in the easy and in the hard task did not differ between groups. Retention in the time between training sessions was not different between TD and DCD groups either. The DCD group improved significantly on all balance tasks.

**Conclusions:**

The findings in this study give a fairly coherent picture of the learning process over a medium time scale (5 weeks) in children novice to active computer games; they learn, retain and there is evidence of transfer to other balance tasks. The rate of motor learning is similar for those with and without DCD. Our results raise a number of questions about motor learning that need to be addressed in future research.

## Highlights

Practice improved the dynamic balance task comparably in TD and DCD.

There is spontaneous transfer of learning in TD children and in those with DCD.

## Introduction

Coordination problems in children with Developmental Coordination Disorder (DCD) are characterized by slow and inaccurate performance of motor skills, including activities of daily life, sports, and leisure activities [1]. The most viable hypothesis to explain impaired motor control in children with DCD, as discussed in the recent literature, is the internal modeling deficit (IMD) hypothesis [2]. According to the IMD hypothesis, children with DCD have a reduced ability to use predictive motor control caused by the lack of a good forward model [3]. An alternative explanation for their poor motor skills could be lack of experience (or practice), since motor performance and activity are reciprocally related [4]. A proportion of children with DCD might not get a chance to experience new motor skills or become proficient through practice due to loss of motivation or lack of opportunity.

The Diagnostic and Statistical Manual of Mental Disorders, Fourth Edition (DSM-IV) published in 2000, stated that the performance of children with DCD in daily activities that require motor coordination, is substantially below that expected given the child’s chronological age and measured intelligence [5]. An important new addition to the criteria for diagnosing DCD is included in the DSM-5 [6], which states that, in children with DCD, the acquisition and execution of coordinated motor skills are substantially below that expected given the individual’s chronological age and opportunity for skill learning and use (see Table 1). The recent European Academy of Childhood Disabilities (EACD) guidelines for diagnosing DCD [7] also include a similar statement to the DSM-5 regarding acquisition of skill [6]. This change in wording acknowledges that deficits in motor learning should be taken into consideration when evaluating performance and that contextual factors play a large role in the delayed acquisition of skilled motor behavior.

**Table 1:** DSM-5 Diagnostic Criteria for Developmental Coordination Disorder [6].

A	The acquisition and execution of coordinated motor skills is substantially below that expected given the individual’s chronological age and opportunity for skill learning and use. Difficulties are manifested as clumsiness (e.g., dropping or bumping into objects) as well as slowness and inaccuracy of performance of motor skills (e.g., catching an object, using scissors or cutlery, handwriting, riding a bike, or participating in sports).
B	The motor skills deficit in Criterion A significantly and persistently interferes with activities of daily living appropriate to chronological age (e.g., self-care and self-maintenance) and impacts academic/school productivity, prevocational and vocational activities, leisure, and play.
C	Onset of symptoms is in the early developmental period.
D	The motor skills deficits are not better explained by intellectual disability (intellectual developmental disorder) or visual impairment and are not attributable to a neurological condition affecting movement (e.g., cerebral palsy, muscular dystrophy, degenerative disorder).

DSM-5: Diagnostic and Statistical Manual of Mental Disorders Fifth edition

Given this fact, remarkably little research is available that has focused on how children with DCD learn new motor skills, especially when compared with the extensive research examining the underlying deficits [3,8]. The few studies that have attempted to study learning in children with DCD are inconsistent in outcome concerning the presence and extent of motor learning problems in this group [9–13].

To date, no studies have been conducted to determine whether children with DCD only need more time and opportunity to practice in order to reach an appropriate level of performance compared to their peers without DCD or whether specific interventions are necessary [14]. If impaired performance in children with DCD is caused by limited experience, then providing the child with more time to practice and increasing the affordances within their home and school environment should suffice to resolve inaccurate or incomplete internal models of movements.

Although one of the DSM-5 criteria states that the motor skill deficits seen in children with DCD are not attributed to a neurological condition affecting movements, recently published reports indicate deviant brain activation in DCD [1,15,16]. Three brain regions have been proposed as possible foci for the aetiology of DCD: (1) cerebellum [17–19], (2) parietal cortex [20,21] and (3) basal ganglia networks [22]. Different patterns of activation in these brain areas have been linked to poor performance across a range of functional activities.

The *cerebellum*, known to be important in (implicit) motor learning, has been suggested as a primary site of dysfunction in DCD. This has been confirmed in fMRI studies where children with DCD demonstrated less activation in various cerebellar regions compared to typically developing (TD) children during a fine-motor task [16]. Another area of the brain known to be an important site for processing sensorimotor transformations, building of internal models and motor learning, the *parietal cortex*, has also been noted as impaired in DCD and is associated with the IMD [21]. Studies showing poor sequence learning [11] and impaired force control in DCD [23] suggest *basal ganglia* to be involved in the motor dysfunction in children with DCD, however these findings have not yet been replicated in fMRI studies. Importantly, given the heterogeneity of individuals with DCD, multiple brain regions should be considered to contribute to the motor coordination problems seen in children with DCD.

Although a few studies have attempted to study motor learning in children with DCD over a brief time period and a limited amount of trials [11,12], there is a lack of research relating to the acquisition of motor skills by extensive repetition. Thus far, the positive effects of intervention indicate that new motor skills may be learned or existing motor skills refined over a longer time period if children are given adequate opportunity for skill use. For example, task-specific cognitive and functional motor skill intervention approaches like the Cognitive Orientation to daily Occupational Performance and Neuromotor Task Training, appear to be effective in children with DCD and recipients of these interventions report that they enjoy these forms of therapy [24,25]. However, details about how the motor skills acquired using these approaches evolve over time, are missing.

Only one study with a very small sample (i.e. two children with probable DCD) examined the rate and process of motor learning over a long time scale of weeks [26]. In this study, learning was examined by observing the change in proficiency after repetitive practice of a hockey slap shot using 1200 trials [26]. The two children with probable DCD (p-DCD) did not improve the kinematics of the movement nor the speed of the hockey slap-shot compared to initial performance of two matched TD children, one with and the other without experience. The children with p-DCD remained extremely variable in timing. Based on these findings, there is a clear need for sound studies using different motor learning paradigms, from simply giving enough opportunity and creating environmental affordances to tailor-made interventions.

The use of computer games has steadily grown as a highly popular form of entertainment. Researchers have progressively explored the application of motion-steered digital games for learning or training purposes in a wide variety of areas (e.g. stroke, obesity, cerebral palsy, DCD). In many countries it is hard to find children that are totally novice to these motion-steered computer games. However, in one of our earlier studies, we observed that many children living in low-income neighborhoods in South Africa have no experience with these kinds of games [25]. This presented a unique opportunity to evaluate motor learning with two novice groups of learners with different motor performance levels, without the bias of confounding factors related to differences in prior exposure.

The most common and oldest approach to learn a motor task is by repetition. This ‘Practice makes perfect’ approach proposes that learners improve a skill by consistently rehearsing it. In the early learning stage, acquiring the topology of the movement is important and at a later stage, the dynamic control of joint coordination gradually emerges while practicing the task [27]. Thus, repeating the same task in a controlled way using a standardized game is a good way to examine motor learning.

Successful motor learning should result in three outcomes: i) improved performance in the trained task; ii) retention of skill over longer time periods; and iii) transfer of the learned skill to a different task with comparable elements. The current study was set up to test each of these three components of learning and whether they would be different between groups of children with and without DCD.

We used performance on a motion steered computer game, the Nintendo^®^ Wii Fit ski-slalom game, to assess motor learning. The children performed the game 50 times in an easy and 50 times in a harder condition, over a period of five weeks. The main measure of performance was the Wii score, which is calculated using the number of gates missed in the slalom and the duration of the run down the ski slope.

The main aim of this study was to examine the rate of learning in children with DCD and an age-matched control group using 100 trials of a motion steered computer game. We aimed to answer the following questions: 1) is the initial level of the game performance different between groups? 2) do children learn differently if the game is easy or difficult? 3), are these effects different for the two groups. We hypothesized that children with DCD would learn less compared to the control group as reflected by a steeper slope in the control group. If children with DCD learn equally as well as the age matched controls, then both learning slopes would run parallel to each other. Therefore, we analyzed if there was a difference between groups in 4) learning rate, 5) retention and 6) if there was transfer to other balance tasks. We expected retention and transfer to be lower in children with DCD. Lastly, we explored the strategies used by the children to achieve higher scores in the game.

## Methods

### Research Design

A pre-post experimental design was used to evaluate changes in game performance during 100 trials of the Nintendo^®^ Wii Fit ski-slalom game in children with and without DCD at two levels of task difficulty.

### Participants

To select participants, we used the same procedure as described in our earlier studies [25,28]. Teachers were asked to assist in identifying children with motor coordination problems based on their observation of the children in class and on the playground. They highlighted the child’s name on a class list of names and returned this list to the researchers. Parents were asked to complete a questionnaire about possible health related problems. The four *DSM-5* criteria were then used to identify children with DCD [6]. All children, aged 6–10 years (Criterion C) who scored below the 5^th^ percentile on the Movement Assessment Battery for Children 2^nd^ edition (MABC-2) (Criterion A), who were identified as having a motor coordination problem by the teacher (Criterion B), whose parents reported no diagnosis of a significant medical condition known to affect motor performance (Criterion D), and whose teacher affirmed the absence of intellectual or cognitive impairment (Criterion D), appeared to fulfill the criteria for DCD. Through this procedure, 18 children were selected to participate in the study and were age-matched with 18 TD children from the same classes.

TD children had: 1) a score above the 16^th^ percentile on the MABC-2, 2) no evidence of functional motor problems as observed by their teacher, 3) no diagnosis of a significant medical condition as reported by a parent and 4) absence of intellectual or cognitive impairment as reported by their teacher. All participating children in both groups confirmed to have no experience whatsoever with motion steered computer gaming when questioned by the researchers.

### Instruments

#### The Movement Assessment Battery for Children-2 (MABC-2)

The MABC-2 [29] consists of eight physical subtests used to assess motor coordination in children aged 3–16 years. Raw scores for each item are converted into standard scores (SS). The Total Standard Score (TSS) is a sum of the individual standard scores, converted to standard score and gives an impression of the overall motor proficiency. Component Standard Scores (CSS) are a reflection of abilities in the three major performance areas, viz. Manual Dexterity, Aiming and Catching and Balance. TSS and CSS may be expressed in percentiles. Scores at or below the 5th percentile (equal to 5^th^ SS) are considered as definitive for motor coordination problems and scores between the 5^th^ and 16^th^ percentile (equal to 5–7 SS) are suggestive of a risk for motor problems [29]. The MABC-2 is considered a reliable and valid measure to assess motor performance [30,31]. In children with DCD, internal consistency is reported to be high (alpha = 0.90) and test-retest reliability for the total scores is regarded as excellent (ICC = 0.97) [29,30]. Since there are no South African norms available for the MABC-2, we used Dutch norms [30]. To be sure that children fulfilled criterion A of the DSM-5, a more rigorous cutoff value (5^th^ instead of the 15^th^) was applied than required by the EACD guidelines for the diagnosis of DCD [7].

#### Wii Fit ski slalom game

The Nintendo^®^ Wii Fit ski slalom game was used according to a comparable protocol developed by Jelsma et al., 2014 [32]. The game requires dynamic balance control whereby players shift their body weight from one leg to the other in a timed manner while standing on a Wii Balance Board (WBB) with Bluetooth wireless connection. When standing on the WBB, the child can steer a virtual character known as a Mii, by shifting their weight. When the child shifts his or her center of mass (CoM) forward or backward, the Mii speeds up or slows down; shifting the CoM to the left and right (laterally) directs the Mii sideways. The sensitivity of the WBB is normalized according to the child’s weight, which is a standard procedure of Nintendo^®^ Wii Fit. The WBB has been shown to be a valid and reliable device to measure the Center of Pressure (COP) compared to force plate data [33].

The goal of the ski-slalom game is to steer the Mii through gates along a slope without missing a gate and as fast as possible. Two versions of the game were played alternately, the easy (19 gates) and the advanced (harder) version (27 gates). Importantly, the spatial layout of the gates on the slope within each version is invariant.

From a motor learning perspective, it is important to be aware of the different sources of feedback available to the player during and after the game. Due to the direct coupling between weight transfer and movement of the Mii, the player is able to make an implicit comparison between the intended movement induced by the weight shift and the actual movement of the Mii (Knowledge of Performance). Moreover, during the game there is immediate visual and auditory reinforcement to signal success or failure as the Mii passes through each of the gates. Immediately after a run, the number of points obtained (Knowledge of Results: the Wii score) is presented on the screen. The Wii software derives a Wii score from the following equation: Wii Score = T + (# x 7s), where T is the time taken to reach the end of the slope, # is the number of missed gates and 7s is the penalty in seconds for each gate missed. Thus, a higher Wii score reflects worse performance.

### Procedure

#### Ethics Statement

Approval for the study was granted by the University of Cape Town, Faculty of Health Sciences Human Research Ethics committee (UCT HREC Reference number: 556/2014), the Western Cape Department of Education as well as by the principal of the respective school. Written informed consent was obtained from all parents and each child according to the declaration of Helsinki. For the selection of the children, a team of qualified physiotherapists who had received additional training on the administration of the MABC-2 prior to commencement of the study was used.

#### Training

Four television monitors and four Nintendo^®^ Wii Fit gaming consoles, including the balance boards were set up in an unused room on the school premises. Four children participated simultaneously on the systems, separated by large boards so they could not see the other children’s consoles, under the supervision and guidance of two trained student therapists. The role of the students during training was to instruct, encourage and motivate the children and document all the scores. Children engaged in 20 minutes of gaming, twice a week for a period of five weeks. The participant’s score on each run was recorded. If children missed a session, they were offered an opportunity to attend a catch-up session, preferably in the same week, if that was not possible they came for an extra session in the next week. All children in the study completed 100 trials.

#### Transfer tasks

As a generalization or transfer task, all children performed eight balance tasks: i) the five balance items as described in the MABC-2, ii) one item of the component Balance of the Bruininks Oseretsky Test of Motor Profiency, second edition (BOT-2) [34], iii) the Wii Fit Yoga pose game with both legs tested. All tasks, except the Yoga pose was executed once before and once after the 5-week training period.

#### Balance items of the MABC-2

To test static and dynamic balance in a standardized way, the five items of age band 2 of the MABC-2 [29] were used as transfer items. The static balance items included standing on an unstable balance board for 30 seconds on each leg and the dynamic balance items, walking toe to heel forwards for 15 steps, and hopping in five squares on the left and right leg.

#### Single-leg stance on balance beam

The one-leg standing position on the balance beam is an item of the BOT-2 [34], in which the child is asked to stand on the beam on a preferred leg, placing their hands on hips. The non-preferred leg is raised with the knee flexed 90 degrees and shin parallel to the ground. The one-leg balance beam task was chosen as a transfer task because it does *not* provide augmented visual feedback and resembles natural circumstances that children might encounter during playground games.

#### Yoga task

The Yoga pose is one of the Wii Fit game options. It is a one-leg standing posture on the WBB, in which the player uses their hands to hold their flexed knee in front of the body. An on-screen instructor demonstrates how to acquire this posture and the software detects the efficacy of the performance. The performer is required to focus on a representation of the postural sway from their COP shown on screen. The time held in balance recorded by the computer (maximum of 30 seconds) and the postural sway represented by the COP are combined to generate a point score with a maximum of 50 points per leg. Both legs were tested. The Yoga task was chosen as a transfer task because both the Wii ski slalom game and the Yoga task require the performer to stand on the Wii balance board and watch the screen on which augmented dynamic visual feedback related to task performance is presented. Moreover, we chose to record this item once (for a maximum of 30 seconds per leg) at every training session between run 5 and 6 to follow the transfer over time from the dynamic training to this static item. An advantage of the way this item is recorded is that it takes postural sway into account. An advantage of using the Yoga pose as a transfer task is that it does not have a large ceiling effect, in contrast with the balance test items of the MABC-2 and BOT-2.

### Enjoyment rating scale

Because children might lose motivation over the long period of training and the large number of trials, we included an enjoyment rating scale to measure the level of enjoyment experienced while doing the game. Children chose from five different smiley faces to rate their gaming experience (0 is “no fun at all” 1 is “boring”, 2 is “a bit of fun”, 3 is “fun” and 4 is “super fun”), using a scale that was developed for one of our earlier studies [32]. We evaluated how much the child enjoyed playing a Wii game on three occasions (after the first week, after 3 weeks and after the last session).

### Data analysis

All data were checked for normality and equality of variances and appropriate parametric or non-parametric analyses were performed. Differences in demographic characteristics and motor tests between the groups were calculated at baseline using Pearson’s Chi squared test (sex and handedness) or t-tests (age, MABC-2 total and cluster scores, one-leg standing on the balance beam (BOT-2) and Yoga score).

For research question 1, to test if the initial level of the game was different between groups, independent t-tests were performed using the data from the initial run of the easy and hard condition in the first training session.

For research questions 2 (main effect of training) and 3 (main effect of task difficulty) and their interactions with group, Wii scores were analyzed in a repeated measure ANOVA with training session (10), task difficulty (2), and runs (5) as within group factors and group as the between factor.

To estimate the relationship between repetitions and the Wii score (research question 4), a linear curve was fitted to the 50 data points of each individual child for the two difficulty levels. The steepness of the slope (β) reflects the extent to which performance improves as training progresses, i.e. the rate of learning. Estimates of slope (β) per child were entered into separate repeated measures ANOVA (task difficulty as within factor, and group as the between factor).

To test the retention effect (research question 5), we compared the Wii-score, on the last run of the previous session with the first score of the following session (9 pre-post scores and two groups), using repeated measures ANOVA.

To test if training transferred to other balance tasks (research question 6), we compared pre and post scores of the transfer items using Paired-Sample Wilcoxon Signed Rank Test. To test the changes of performance on the Yoga task, repeated measures ANOVA was performed (within factor task with 10 repetitions, and group as the between factor).

To examine possible differences in strategies exploited to get higher Wii scores, we used both the individual regression outcomes based on the number of gates missed and based on the actual time needed to finish each consecutive run into account. We anticipated the following strategies to significantly improve the score and checked if they would distinguish between groups of children: A) Reducing speed to such an extent that fewer gates are missed (Speed accuracy trade-off strategy); B) Missing fewer gates by improving accuracy without changing time (Accuracy strategy): C) Missing fewer gates and significantly speeding up by leaning forwards (Mastery Strategy). Differences between groups in the frequencies of the strategies were tested using Pearson’s Chi squared test. The scores of the enjoyment rating scale were reported using descriptive statistics.

Significance level was set at *p* < .05. All statistical analyses were run in Statistical Package for the Social Sciences (SPSS Inc., version 22). Only significant interactions and those that involve the factor group were reported.

## Results

### Group comparability

One child from the DCD group was taken ill with meningitis at the beginning of the study and was subsequently withdrawn. Another child from the TD group missed several training sessions due to absenteeism from school and therefore failed to complete the study. The results of these two children have not been included in the analysis.

Children with DCD (n = 17) were not different from TD children (n = 17) in terms of age (*t* = 0.74, *df* = 32, *p* = 0.64; TD 7.65 (SD 1.1), DCD 7.94 (SD 1.2) and gender (χ^2^ = 0.11, *p* = 0.75; 9 boys and 8 girls in each group).

All children with DCD scored below the 5^th^ percentile on the MABC-2. As expected, the mean TSS was significantly lower than for the TD group (*t* = -11,33, *df* = 32, *p* = 0.001) (For means see Table 2). The groups also differed on the cluster score Manual Dexterity (*t* = —6,73, *df* = 32, *p* = 0.001), Catching and Aiming (*t* = -2.14, *df* = 32, *p* = 0.042) and on the Balance Component (*t* = -9.57, *df* = 32, *p* = 0.001); seven children scored between the 5^th^ and 16^th^ percentile and ten children scored at or below the 5^th^ percentile, whereas all TD children scored above the 16^th^ percentile on the Balance Component. Lastly, the item standing on a balance beam (BOT-2), showed a trend towards differences between the two groups on pre-test values (*t* = -2.06, *df* = 16.59, *p* = 0.055, corrected for unequal variance; TD 9.9 (0.33) DCD 8.6 (SD 2.4).

**Table 2:** Means (SD) of the Movement ABC-2 Cluster and Total Standard Scores of both groups at the start of the study with differences between groups.

Group	Manual Dexterity	Aiming and Catching	Balance	Total Standard Score
DCD (n = 17)	5.24** (2.6)	5.94* (3.4)	5.00** (1.7)	3.71** (1.4)
TD (n = 17)	11.94** (3.2)	8.00* (2.0)	11.47** (2.2)	11.06** (2.3)

** significance p < .001,

* significance p < .05.

At the start of the training period, children with DCD scored lower on the Yoga task compared to TD children (Left leg: DCD 14.60 points (SD = 18.91); TD 28.53 points (SD = 17.48) *t* = -2.22, *df* = 32, *p* = 0.03; Right leg; DCD 17.82 points (SD = 18.83); TD 32.12 points (SD = 15.22) *t* = -2.43, *df* = 32, *p* = 0.02).

### Initial performance at the first training session

**Fig 1:**
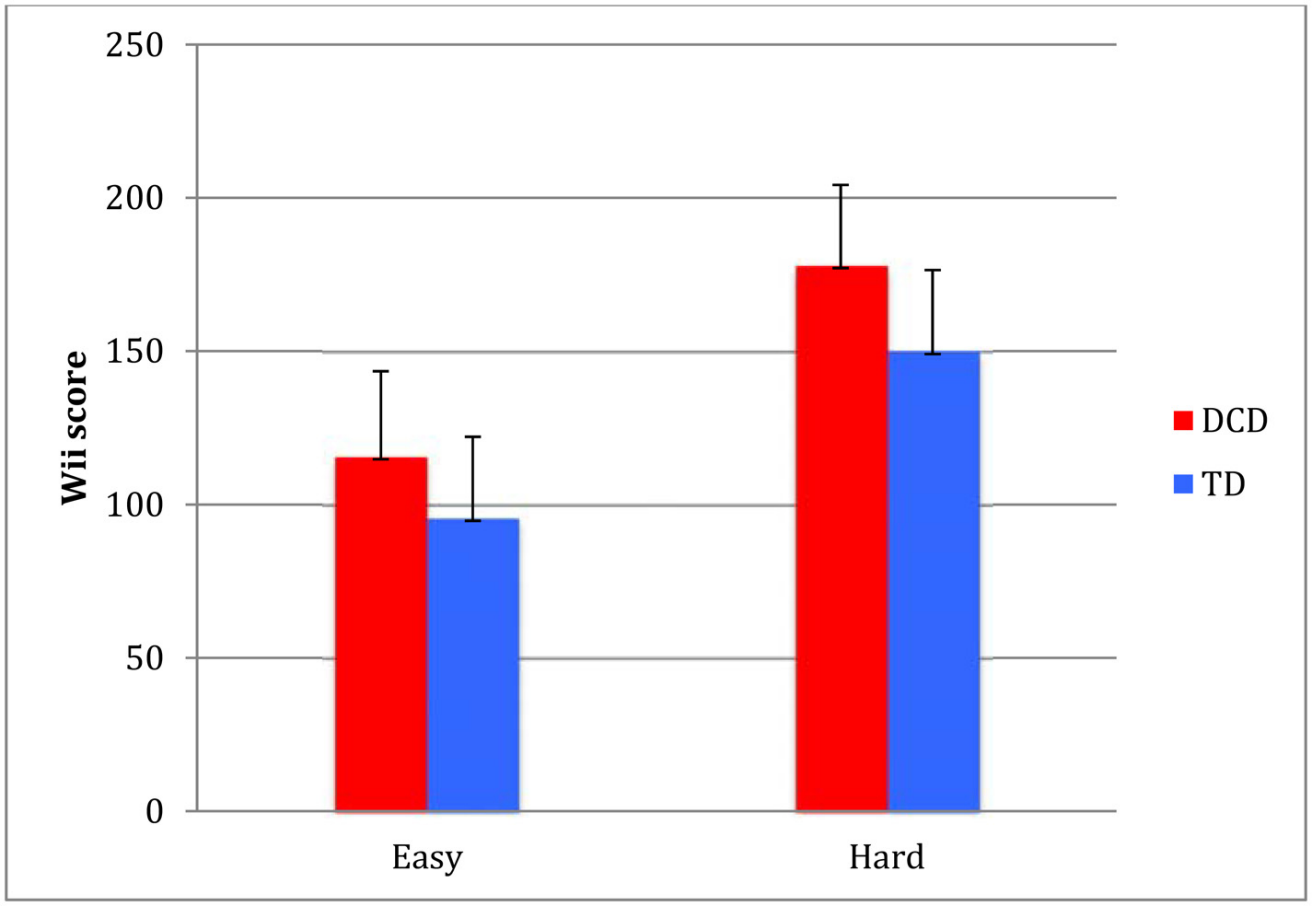
Initial Wii scores for the easy and hard condition (error bars represent SD). A lower score indicates better performance.

As depicted in Fig 1, the initial level of proficiency (first run of the easy and the hard task during the first training session) as determined by the Wii score was different between the DCD and TD groups (easy TD 95.7 (SD 21), DCD 115.8 (SD28), *t* = -2.37, *df* = 32, *p* = 0.024; hard TD 150.1 (SD 26) DCD 178.0 (SD 26) *t* = -3.10, *df* = 32, *p* = 0.004).

### Motor learning

**Fig 2:**
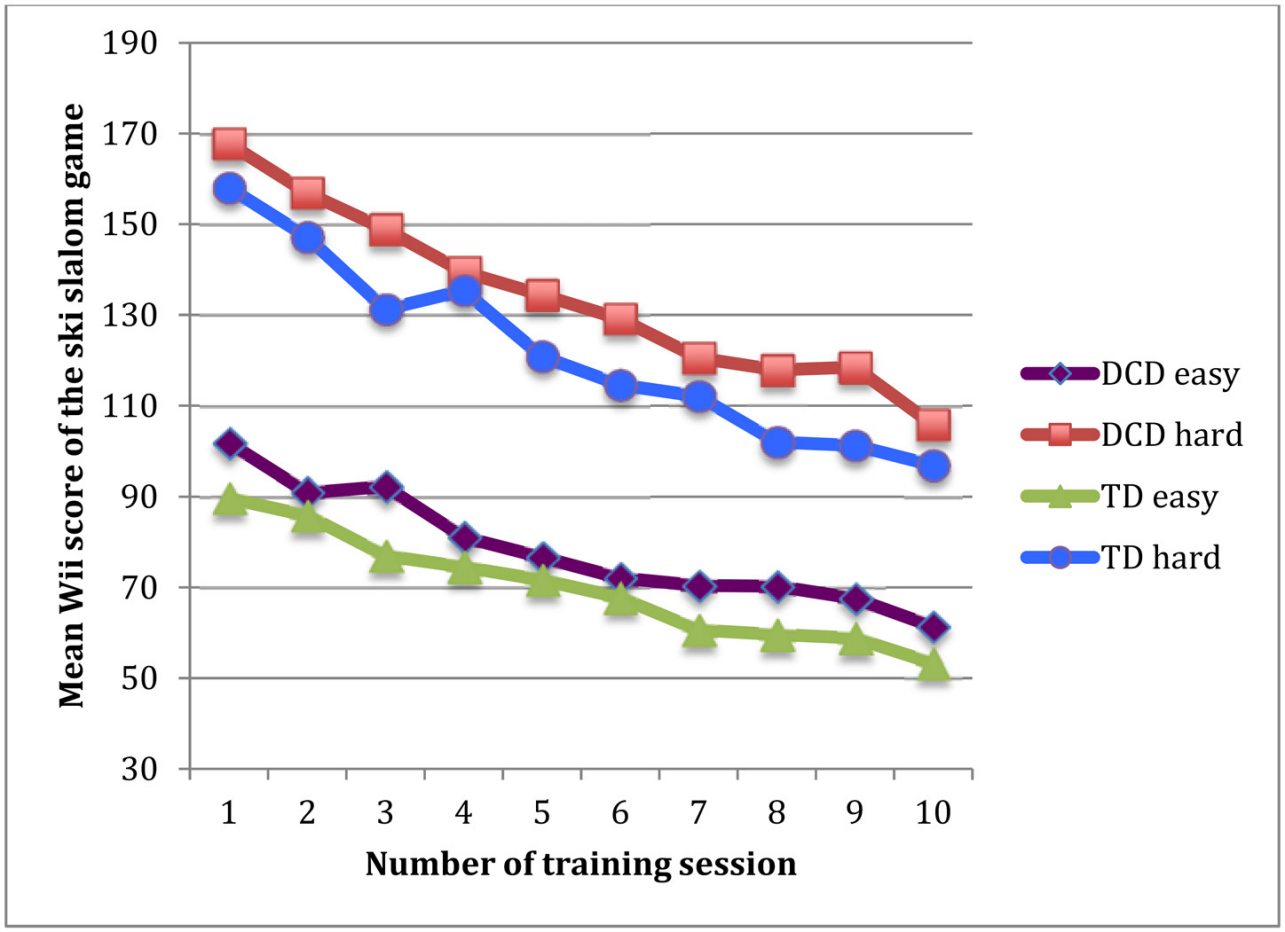
The mean Wii score of the easy and hard version of the ski slalom game of both groups over the ten training sessions.

Next, we analyzed changes in the Wii-score over 50 runs per condition of task difficulty. Both groups of children improved their Wii-score, confirmed by a main effect of training (*F*(9, 24) = 20.29, *p* = 0.001, *η* ^2^ = 0.88). The task difficulty effect was very robust (*F*(1, 32) = 1058.79, *p* = 0.0001, *η* ^2^ = 0.97) as was the run effect (*F*(4, 29) = 45.20, *p* = 0.001, *η* ^2^ = 0.86), the latter indicating improvement in performance within each training session over the five repetitions of the same task. Importantly, the mean difference between the two groups in the effect of training was not significant (*F*(1, 32) = 2.56, *p* = 0.12, *η* ^2^ = 0.07) (Fig 2).

The steepness of the slope (β) reflects the *rate of learning*, i.e. the extent to which performance of each *individual* child became better as training progressed. All TD children and all children with DCD, except one child, improved their Wii score significantly over the two sets of 50 trials. The mean steepness of slope was not different between the groups (*F*(1, 32) = 0.04, *p* = 0.85, *η* ^2^ = 0.001). The mean slope in the easy task was less steep (β = -0.42) than in the difficult task (β = -0.67) indicating that rate of learning during the easy task was lower (*F*(1, 32) = 73,68, *p* = 0.0001, *η* ^2^ = 0.70). Notably, the effect of task difficulty on the slope was not different for the two groups (*F*(1, 32) = 0.73, *p* = 0.40, *η* ^2^ = 0.02).

However, the Wii-score is a composite score that takes speed and accuracy into account. Hence, we also plotted the individual slopes for the gates missed. All children significantly decreased the number of gates they missed (p<0.05) except one (the same child with DCD that did not show improvements on the overall Wii score; *p* = 0.98). If a child used more time, but less than 7 seconds, to finish the run and if this enabled him to miss one gate less, the overall Wii-score still improved.

**Fig 3:**
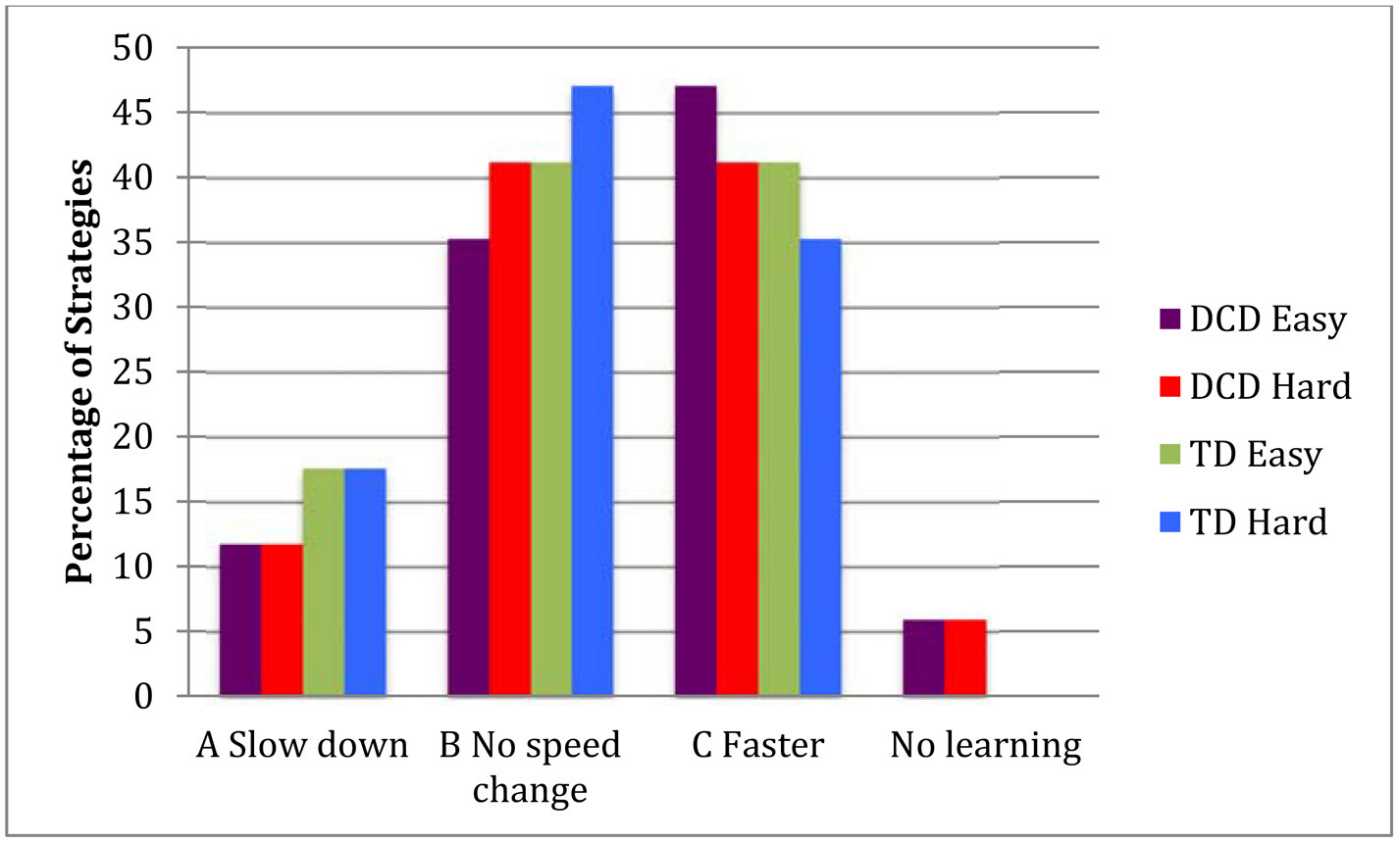
Strategies used to miss fewer gates and improve the Wii score.

Analyzing the different strategies of improvement, it became clear that all three strategies (see Method-data analysis) were used. Five children (2 DCD and 3 TD) used Strategy A to achieve a better score by slowing down and missing less gates, in both sets of 50 trials. Of the remaining children, half used Strategy B only by improving accuracy, and the other half used Strategy C, the most complex strategy. This last group was able to significantly increase speed and accuracy through extended practice. This analysis based on the individual data indicates that all options to improve the scores were utilized by both groups of children. Slowing down (strategy A) was clearly the least used. Notably, no predominant strategy was seen for children with and without DCD (χ^2^ = 0.63, *p* = 0.73) (Fig 3).

### Retention

**Fig 4:**
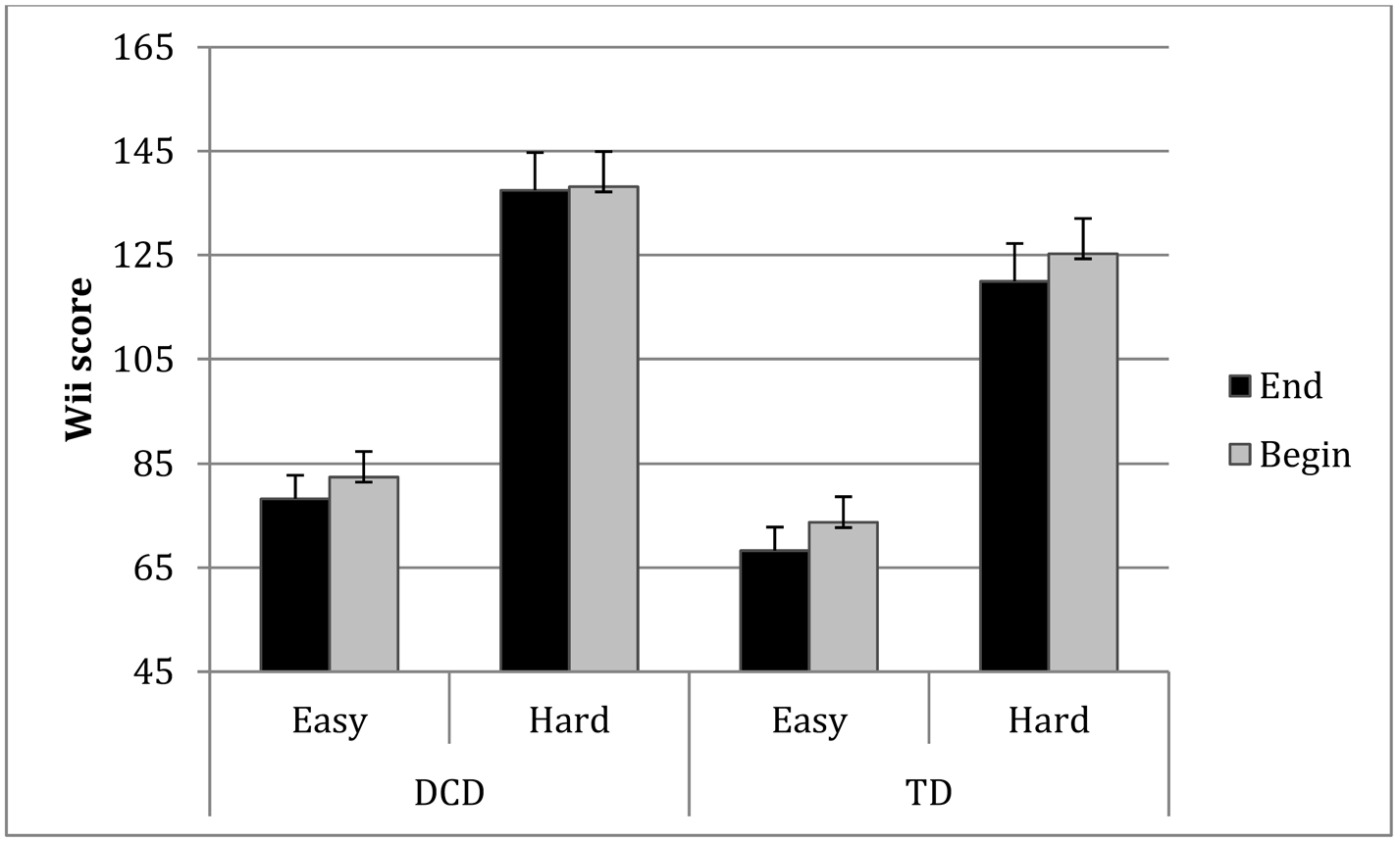
The mean score of the last run in the previous training session and the first run of the next training session of both groups per task condition.

For this analysis we compared the Wii-score on the last run of the previous session with the first score of the following session (9 pre-post scores). The time between the consecutive training sessions ranged from two days (within the school week) to four days (over the weekend), but was similar for both groups. The mean score of the last run in the previous training session was significantly better than for the first run of the next training session (*F* (1, 32) = 26.43, *p* = 0.0001, *η* ^2^ = 0.45). This decrease in performance was not significantly different between the two groups, but there was a trend for the TD children to poorer retention (Fig 4), (*F* (1, 32) = 3.76, *p* = 0.06, *η* ^2^ = 0.11). Moreover, the interaction between training session and retention score was significant (*F* (8, 25) = 2.81, *p* = 0.02, *η* ^2^ = 0.47). Further analysis showed this was a quadratic effect *(p* = 0.03). In the beginning (when scores were still poor) the differences between the last and the first score of the following session were small. In the middle of the training, differences became larger and by the end of the training period, the differences became smaller again. No interaction with group emerged *(p* = 0.39).

### Transfer

The DCD group improved on all balance items (Table 3). The TD group only showed better performance on two items because of the ceiling effect of the other items., all TD children reached the maximum score on the three balance items (i.e. Walking on a line, hopping on the best leg and standing on a balance beam).

**Table 3:** Mean pre- and posttest scores of the MABC-2 balance items and the BOT-2 balance item with p-values.

Balance items	DCD pretest (SD)	DCD posttest (SD)	p-value	TD pretest (SD)	TD posttest (SD)	p-value
MABC-2 Standing on Best Leg (sec)	13.6 (9.3)	21.5 (10.0)	**.*006*** **	22.7 (8.8)	26.9 (6.0)	**.*05***
MABC-2 Standing on Other Leg (sec)	9.1 (7.2)	14.9 (9.4)	**.*023*** *	18.4 (9.8)	19.7 (8.6)	.*506*
MABC-2 Walking over a line (#steps)	8.7 (5.2)	14.5 (2.2)	**.*001*** **	13.3 (3.9)	15 (0)	**.*042*** *
MABC-2 Hopping Best Leg (#hops)	3.9 (1.3)	4.7 (0.9)	**.*005*** **	4.8 (0.4)	5 (0)	.*083*
MABC-2 Hopping Other Leg (#hops)	2.4 (1.9)	4.1 (1.2)	**.*006*** **	4.4 (0.9)	4.9 (0.3)	.*054*
BOT-2 Standing on balance beam (sec)	8.7 (2.5)	9.9 (0.5)	**.*042*** *	9.9 (0.3)	10 (0)	.*157*

** significance p < .01,

* significance p < .05.

**Fig 5:**
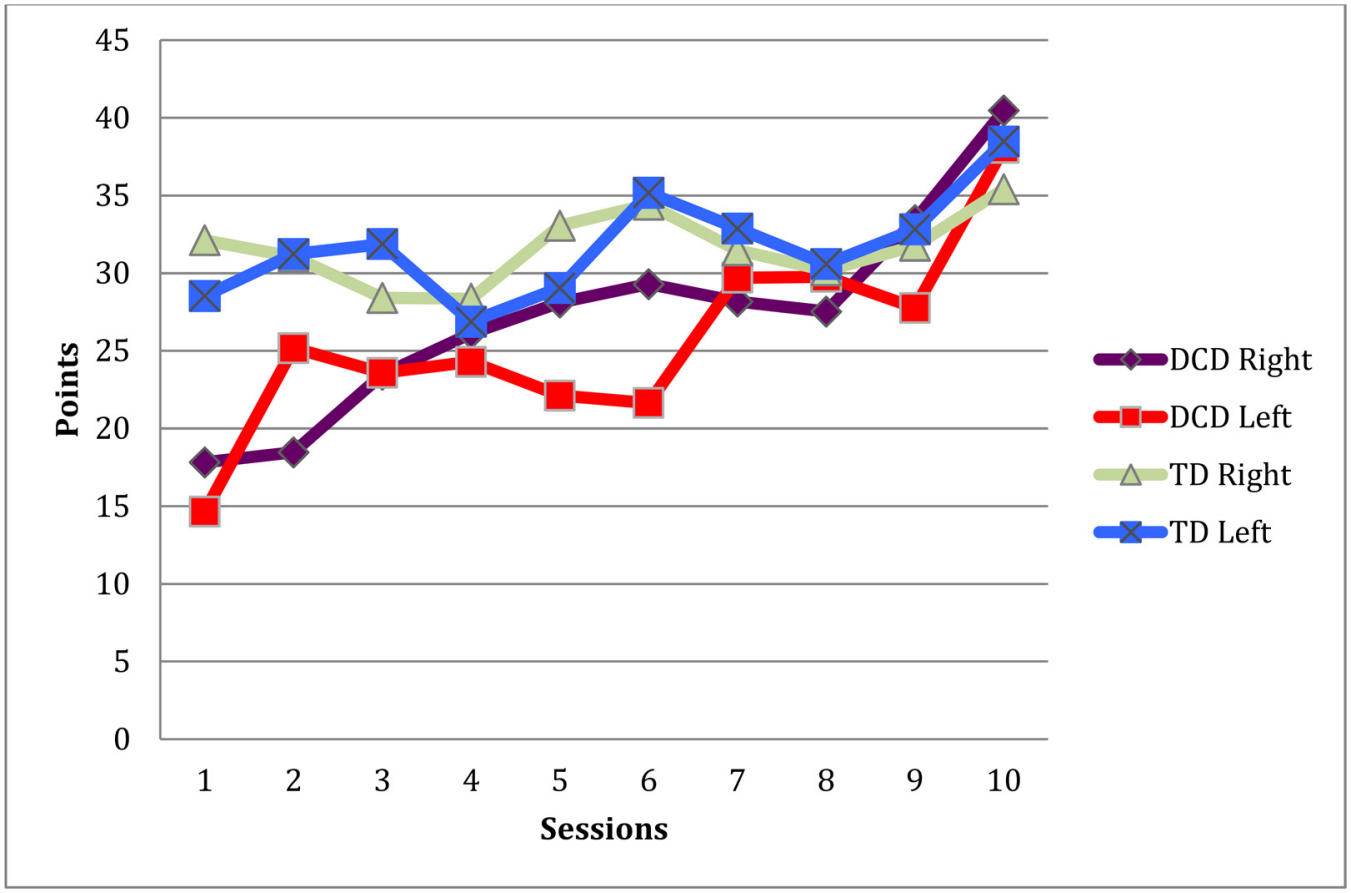
Mean number of points on the yoga task per leg per training session in TD children and children with DCD.

Performance on the Yoga balance task improved over the 5 week period (Left leg (*F*(9, 24) = 4.22, *p* = 0.02, *η* ^2^ = 0.61; Right leg (*F*(9, 24) = 4.53, *p* = 0.03, *η* ^2^ = 0.49). After the training, the groups no longer differed on these tasks. The data depicted in Fig 5 shows that the DCD group caught up with the TD group in duration and stability of standing on one leg.

### Enjoyment rating scale

After the first week, 31 children reported the training to be “super fun”, two children rated the training as “fun” (both TD) and one child rated the training as “a bit of fun” (DCD). After five weeks and playing the game a 100 times, only five children changed their opinion. Two increased their score by one point to “super fun” (TD) and 2 decreased their score with one point to “fun” (1TD, 1 DCD) and one by two points to “a bit of fun” (DCD).

## Discussion

The DSM-5 highlights poor skill acquisition as a key feature in the description of DCD. Therefore, the general aim of this study was to systematically explore the acquisition of a new motor skill in children with and without DCD. More specifically, we examined how learning evolves over 100 trials of a single task, performed over five weeks. The main results indicate that independent of task difficulty, the children with DCD 1) do not differ from TD children in their rate of learning; 2) have similar levels of retention; and 3) show evidence of transfer to dynamic and static balance tasks.

In this study, children with and without DCD played 10 runs of the ski slalom game twice a week over a five-week time period. The experimental manipulations we introduced were effective, as repetitive training improved Wii-scores, both within and between sessions, and increased task difficulty reduced the mean Wii-scores. Remarkably, all children remained motivated throughout the five-week study although the same task was repeated many times. The important finding of this study is that both groups improved their performance comparably and used similar strategies to improve their scores. Children with DCD did not show a retention deficit, that is, children in both groups lost a comparably small percentage of their score in the two to five days between sessions.

Based on these data, it is clear that children with DCD do not have a learning deficit in the first stages of learning. An important key to this finding may be the augmented feedback that is presented consistently and in large doses via the Nintendo^®^ Wii Fit interface. Ten training sessions of 20 minutes duration provided enough practice opportunity for all children (except one) to significantly improve their scores; the gain in Wii-score was on average about 35%. This outcome is in line with results of most intervention studies, which have shown that intensive, task-oriented therapy improves motor skills [8,24,25]. Another important finding is that the rate of learning does not seem to plateau after 100 runs, which implies that there is room for improvement.

### Lack of differences between groups in the rate of learning

The learning curves of children with DCD were similar to those of the TD children, despite having poor scores on the balance items and the total score of the MABC-2. So what could explain the often-reported motor learning deficit in children with DCD? Could it be just a matter of lack of practicing motor skills? For the very early stages of learning (the first 100 trials) our data suggest that exposure to training opportunity, and knowledge of performance and result may best explain the increase in level of proficiency in the game and not the overall motor proficiency. We cannot say if (or when) performance of the two groups will diverge at a later stage. Unfortunately, we could not train the children for a longer time, as training was conducted in school hours and this would have impacted on their classroom attendance.

### Learning the task

An active computer game was chosen for learning by repetition. The ski-slalom game also includes error-based and reinforcement learning since the movements of the child are projected as the movement of the avatar, and success and failure is seen on the screen and heard as an auditory feedback signal. In addition, information about the efficacy of the movements is given at the end of the game as a total score. The freedom to explore different movement strategies within the task, repeating the task at different levels with these large amounts of motivating feedback in the fast changing context of the game, resulted in significantly improved performance in scores for all the children (except one).

A critical question that remains is whether and how much further improvement would be realized in each of the groups if the training program continued. Would the newly learned skill eventually reach the same level in both groups or would the DCD group plateau before the TD group? Our enjoyment rating scale indicates that children generally loved the gaming experience and were still motivated to play the game at the end of the training. It would therefore be possible to extend the number of trials and future studies might use more repetitions to reveal if the groups reach different asymptotes.

We found one study that trained two children with p-DCD on a hockey slap shot using a large number of repetitions [26]. At the end of the training, the kinematics of the movement of the two children with p-DCD still showed a lower level of movement consistency and less efficacy compared to the two TD children, one with and one without hockey experience. This is unlike the lack of difference in our study. Obviously, a hockey slap shot is a very different whole body movement compared to playing a motion steered computer game in which timed weight shifts are required. Moreover, we only looked at the improvements in the Wii performance and did not analyze the underlying kinematics in our study.

Motor learning is a complex process. Ideally, it should have the following three effects: 1) improved performance on the task trained; 2) retention of skill over time; and 3) transfer of learned skill to a different task with comparable elements. Our study included all three aspects, which makes this a robust study of motor learning. Children with DCD did not differ from TD children on either of these aspects.

### Transfer of learning

Eight transfer tasks were used in our study, 6 items taken from general motor tests and the Yoga tasks (performed on both legs). Children in the DCD group improved on all these balance tasks without being specifically trained, while for the TD children, the effect was only significant in two items. This lack of improvement is caused by the fact that both the items of the MABC-2 and BOT-2 have strong ceiling effects in the balance items (see lack of variance in Table 3). Once a child can hop 5 times on one leg or stand on a balance beam for 10 seconds there is no room to measure improvement.

Standing in the Yoga position on one leg was not part of the repetitive training, but the posture was evaluated 10 times over 5 weeks. This means that if children reached a maximum score in all the 10 attempts recorded, they would have practiced this task for 5 minutes per leg over the 5 weeks of training. Both groups improved significantly on this task showing it to be the more sensitive task, since it takes postural sway into account. The transfer tasks taken from general motor tests were only measured before and after the whole training period to avoid a possible effect of repeating the task. Here too, the time that children held a one legged stance increased. Evidently, one leg stance improved in conditions with and without augmented visual feedback. This makes it likely that the improvements obtained during the Wii training do indeed transfer to a non-trained task with comparable balance elements. Previous studies on the effectiveness of exergames also demonstrated transfer to other related skills [25,32,35]. The occurrence of transfer of learning raises the question of what is actually learned and questions to a certain extent the notion of task specificity. The Mii is an indirect form of self-observation, which helps a child to learn skills, possibly through activation of the mirror neuron system. The predictive route planning requires frequent timed shifts of weight to steer the Mii whilst not losing balance, which may have lead improved dynamic and static balance control. Specifically, the observed dynamic visualization of the weight transfer may have assisted in acquiring the skills [36].

Since it is has been shown that the amount of transfer or generalization is usually small [36,37] it is important to know which tasks transfer to one another and when tasks do not generalize [38], especially if exergames are used as intervention tools.

### Motor learning deficit in children with DCD

Since all the children in this study were novice to exergames, they are considered to have progressed through the initial stages of learning during the 5 weeks of training. According to Fitts and Posner [39] and Bernstein [40], the first phase of learning is the discovery of one or more effective solutions to controlling the Mii. This should converge to the selection of a task strategy that is effective and feasible for the child, following several trials, depending on the complexity of the task. During these initial stages, we found no differences between the two groups of children in their motor learning abilities whether the tasks were easy or difficult. In addition, no differences were observed between groups regarding the strategies employed to improve performance.

Since it is commonly accepted that children with DCD have a motor learning deficit, what could explain this discrepancy of the present findings and the literature [9–13]? We hypothesize that there could be three related possibilities:

Our first, and most likely, hypothesis is that differences in motor learning will only emerge in the later phases of the learning process. It might be that lack of predictive control, as required in the later stages of motor learning (not reached yet in the current study), will become a problem because of the reported internal modeling deficit [3,8,41]. As a consequence, these children may continue to depend on the cognitive and attention demanding processing of feedback, which was abundantly available in the game. Children may fail if task constraints increase in complexity or during fast paced activities of daily living requiring a level of automatization. In this case, the lack of difference between groups could be explained by the fact that we did not study their motor learning beyond the first stages of learning.

A second possibility could be a meta-cognitive one. In this explanation, the lack of difference between the groups might be unique to the specific task chosen for this study. It has been shown that children with DCD have more difficulties in planning complex skills and evaluating their performance [42]. If the child is not able to implicitly or explicitly analyze what went wrong, it is hard to learn from mistakes. Therapy forms that deconstruct the task into components, clarify details (planning), train the best sequence and increase awareness of the differences between required and actual behaviors can improve the child’s insight into the motor problem [43]. The task chosen for this study is basically a context driven task, which mainly requires trajectory planning and a timed adaptation of direction (based on shift of weight) to accomplish the task, unlike the more complex sequence of planning of actions like in getting dressed or packing a bag.

The third option that may compromise the motor learning capabilities of children with DCD, not measured in the current experiment, is based on psychological factors. In our study all children started at the same level of experience in the task (novice). In daily life, learning a new skill or becoming more proficient at an existing skill requires practice. If you have poor motor coordination, mastering a skill takes more time, more trials and more failures are inevitable. This makes learning or mastering a task less enjoyable and children may feel uneasy in the presence of others when they are unsuccessful. Feelings of failure, depression or anxiety are known amongst children with DCD [44]. In the present study the children had exactly the same amount of training (20 minutes time on the task) and augmented feedback, and they enjoyed the training over the 5-week period. Hence, it is important to find out why children with DCD develop those feelings of failure [44], tend to spend less time than others doing physical activity and seem to be easily discouraged by barriers to physical activity [45]. Is it their social environment, i.e. negative feedback from parents, teachers and peers, or is it the extra effort they are required to make when learning a new task that does not seem to pay off that makes them give up?

In daily life, children may compare themselves to other children and poor performance may easily lead to demotivation and result in lack of practice. In our study, children played games individually, not in competition. This may have been an advantage because they were less afraid of being embarrassed by failure during trials. Ashkenazi [46] reported that children exerted more effort when playing an exergame with their parents, but this was only after they had been training for five sessions giving them a head start without a competitive component. Thus, if we find poorer skills in children with DCD in situations where there is enough opportunity for skill learning, lack of experience by not taking part may be a more likely explanation for the differences with the TD children than a motor learning deficit and should be taken into account in future studies. However, our results do not suggest that lack of experience is a cause of poor motor functioning, nor that poor motor functioning is a cause of not participating. It does show that given the opportunity under optimal feedback and under encouraging circumstances, children with DCD would start to learn the skills comparably.

### Active computer games yes or no?

Research from our group [25,32] and others [36,46] has indicated that the use of virtual environments can be beneficial to the efficiency and outcome of a rehabilitation program, not only for children with DCD but also for those with other medical conditions or lower level of physical fitness [47,48]. Moreover, it has been reported in many studies that children with DCD are less inclined to engage in physical activities [49], so stimulating them through active computer games, which they enjoy, may help them develop or promote some of their skills.

Whether the positive effects of exergaming generalizes to movement proficiency in the daily physical environment needs to be demonstrated. There is currently too little evidence regarding the relationship between fundamental movement skills proficiency and exergaming [50]. Further research should explore whether prolonged exergaming could help children learn certain skills and if this leads to adaption to environmental constraints in the physical world.

### Strengths and Limitations of the study

In this study, we explored motor learning over a five week period in which 100 repetitions took place, which was more extensive than in former publications of procedural motor learning [12,51,52]. Despite the numerous repetitions, the children remained motivated to play the game repetitively, regardless of their level of motor performance. Importantly, we only included children who were naïve towards motion steered computer games in order to make a comparison of children when learning a new motor task without negative experience in earlier attempts to master the task.

A limitation of this study is the absence of norms of the MABC-2 for South African children. To be sure of their low level of motor performance, children were only included in the children with DCD group if teachers indicated them to have motor problems in daily life and they had a motor test score below the 5^th^ percentile. A second limitation of the study is the limited background information of comorbidities in both groups other than the answers to the parent and teacher questionnaires. A third limitation of the study is the relatively small sample size. However, we collected 100 data points per child for the learning curves, which makes the learning curves reliable. Similar findings in other groups of children are needed before one can generalize these results.

## Conclusion

It is frequently hypothesized that the poor motor performance of children with DCD is caused by a deficit in motor learning. In this study, a fairly coherent picture emerges of intact learning processes on a short to medium time scale (5 weeks) in children novice to active computer games; they learn, retain and there is transfer of learning. Of interest, is that learning curves and retention were comparable for novice TD children and children with DCD.

To date, there are too few studies to confirm a motor learning deficit as the main cause for poor motor function in children with DCD. There is a need for longitudinal studies over a longer time scale on a variety of tasks to see if the learning curves will differentiate, to determine when tasks become automated and to establish if capacity of motor planning or anticipatory control determine the final level of acquisition of the skill.

Our findings suggest that the use of active video games may have the potential to be a valuable additional tool in intervention. In this study we focused on a game requiring balance but other games could be used that focus on other skills. Research addressing the additive value of training tasks in a virtual context to current intervention programs is needed.

## Supporting Information

S1 File Wii scores. (XLSX)
